# Longitudinal psychological well-being in caregivers of young children with cleft lip and/or palate

**DOI:** 10.1093/jpepsy/jsaf029

**Published:** 2025-05-07

**Authors:** Nicola M Stock, Debora Blaso, Paul White, Laura Shepherd, Bruna Costa, Karine Edme, Richa Aspland, Matthew Hotton

**Affiliations:** Centre for Appearance Research, University of the West of England, Bristol, United Kingdom; Centre for Appearance Research, University of the West of England, Bristol, United Kingdom; Centre for Appearance Research, University of the West of England, Bristol, United Kingdom; Trent Cleft Network, City Hospital Campus, Nottingham University Hospitals, Nottingham, United Kingdom; Centre for Appearance Research, University of the West of England, Bristol, United Kingdom; Cleft NET East, Addenbrooke’s Hospital, Cambridge, United Kingdom; Cleft NET East, Addenbrooke’s Hospital, Cambridge, United Kingdom; Oxford Institute of Clinical Psychology Training and Research, Oxford, United Kingdom

**Keywords:** caregiver, psychological well-being, cleft lip and palate, screening, early intervention, multidisciplinary care, The Cleft Collective

## Abstract

**Objective:**

Caregivers of children with chronic conditions can experience psychological distress and an impact on quality of life (QoL). Cleft lip and/or palate (CL/P) is one of the most common congenital conditions worldwide. Utilizing data extracted from The Cleft Collective cohort studies in the United Kingdom, this study investigated longitudinal psychological well-being in caregivers of young children with CL/P, to inform screening practices and early intervention.

**Methods:**

Baseline (post-diagnosis) and 5-year questionnaire data were extracted for 525 caregivers (342 biological mothers, 183 fathers/partners). Outcome measures included the PedsQL-Family Impact Module, the Perceived Stress Scale, and the Hospital Anxiety and Depression Scale.

**Results:**

QoL significantly improved from T1 (post-birth) to T2 (5 years) as reported by mothers and fathers/partners. At T2, scores on all measures were aligned with, or more favorable than, norms. A minority continued to report clinically significant levels of distress at 5 years. Predictors of poorer outcomes on all measures included a less positive life orientation, more negative appraisals of CL/P, less favorable baseline scores, lower healthcare satisfaction, and prior mental health conditions. Outcomes were also less favorable for caregivers of children with combined cleft lip and palate compared to other cleft types. Reductions in negative appraisals of CL/P were significantly associated with improved QoL over time.

**Conclusions:**

QoL and psychological well-being in caregivers is generally positive at 5 years. A minority experienced poorer outcomes and routine assessment by a multidisciplinary team is therefore recommended. Targeting early negative appraisals may help to facilitate long-term caregiver adjustment.

Caregivers of children with chronic conditions commonly experience psychological distress in response to their child’s diagnosis and treatment. Stressors may relate to early healthcare interactions, key developmental transitions, treatment burden, and/or changes in the child’s health or the need for hospitalizations (Melnyk et al., 2001). Caregivers have reported elevated levels of stress, anxiety and depression, as well as poorer physical health than caregivers of unaffected children (Bayer et al., 2021; Cohn et al., 2020; Cousino & Hazen, 2013). Prior research has therefore indicated the importance of examining subjective well-being (how people think and feel about themselves and various aspects of their lives; Department of Health, 2012) among caregivers.

Cleft lip and/or palate (CL/P) is one of the most common congenital conditions worldwide, with approximately 1,000 live births per year in the United Kingdom (CRANE, 2019). A cleft is a gap in the upper lip and/or the roof of the mouth, which occurs in utero. Surgery to close the cleft(s) typically occurs during the first year of the child’s life. As the child grows, common issues include a visibly different appearance, speech and language development, and/or hearing difficulties. In the UK, cleft care is delivered by 15 specialist multidisciplinary teams (MDT), located according to regional need. Core MDT members include nurses, surgeons, speech and language therapists, dentists, orthodontists, and specialist psychologists (NHS England, 2013).

Following a diagnosis, caregivers commonly experience a range of conflicting emotions and concerns as they process the long-term implications of their child’s condition (Stock et al., 2024). The reactions of others, including friends, family, health professionals, and members of the public may be perceived as stigmatizing and insensitive (Feragen et al., 2017; Nelson et al., 2012). Feeding difficulties can be particularly distressing for mothers and bonding insecurities have been reported in some studies (Lindberg & Berglund, 2014; Madhoun et al., 2021). Interaction quality, family activities, and family cohesion may also be negatively impacted during this time (Macho et al., 2017).

Previous cross-sectional research has identified a number of factors that may impact how well families adjust to a diagnosis of CL/P. Background factors, such as ethnicity (Crerand et al., 2015), socioeconomic status (Dabit et al., 2014), a prior mental health condition in the caregiver(s) (Stock et al., 2020), recent stressful life events (Stock et al., 2020), caregiver employment insecurity (Stock et al., 2020; Yuan et al., 2022), and caregiver(s) being older at the time of diagnosis (Johns et al., 2018; Stock et al., 2020) may be helpful for contextualization. Child-related factors found to affect parental psychological well-being include cleft type (Thompson et al., 2021), the child having an additional condition(s) such as a syndrome (de Cuyper et al., 2019; van Dalen et al., 2021), and behavioral difficulties in the child (van Dalen et al., 2021). Healthcare-related factors including health insurance status (Crerand et al., 2015), timing of the diagnosis (Johns et al., 2018), feeding difficulties (Madhoun et al., 2021), surgical status (Crerand et al., 2015), burden of care (Cassell et al., 2014), and healthcare satisfaction (Stock et al., 2020) have also been found to influence parental well-being. Finally, psychosocial factors have included parental hope/optimism (Stock et al., 2020; Yuan et al., 2022), parental coping styles (Baker et al., 2009; Yuan et al., 2022), and social support, including close friendships and relationship satisfaction (Baker et al., 2009; Stock et al., 2020).

Historically, little longitudinal research has been conducted in CL/P. Two studies from Norway (Nes et al., 2014) and Japan (Sato et al., 2021) examined psychological well-being among parents of children with congenital conditions, including CL/P. However, many factors pertinent to cleft care were not considered, fathers were not included and data collection ended at or before 3 years of age. Neither study comprehensively investigated the factors contributing to psychological adjustment, which is critical for the identification of parents at risk and the application of psychological intervention.

In 2012, a longitudinal research study entitled “The Cleft Collective” was established in the UK (Davies et al., 2024; Stock et al., 2016). Biological samples and parent/patient-reported questionnaire data have been collected since December 2013. The goals of The Cleft Collective cohort studies are to investigate the biological and environmental causes of CL/P, the best treatments for CL/P, and the psychological impact of CL/P on those affected and their families.

The aim of the present study was to investigate longitudinal psychological well-being in caregivers of young children with CL/P, to inform screening practices and early intervention within cleft services. Specifically, the study aimed to address three primary research questions: (1) How does caregiver well-being compare to general population normative data at age 5 years? (2) How does caregiver well-being change over time (baseline to 5 years)? (3) What baseline factors predict caregiver outcomes at age 5?

## Methods

### Procedure

Ethical approval to establish The Cleft Collective was granted by the South West Central Bristol ethics committee (13/SW/0064). Global Research and Development (R&D) approval was provided by University Hospitals Bristol. Local R&D approvals were obtained from each NHS Trust. Caregivers (biological mothers and their partners) were approached in the clinic by research-trained staff and provided with verbal and written information about the study. Informed consent was obtained from each participating member of the family. Participants completed The Cleft Collective baseline questionnaire (T1) in the period between receiving their child’s diagnosis and their child’s primary surgery and returned their data anonymously via post to The Cleft Collective research team. Participants completed a comparable questionnaire pack when their child was 5 years old (T2). Institutional ethical approval to analyze a subset of the data for the purpose of the present study was obtained from the Faculty Research Ethics Committee at the University of the West of England. Confidentiality agreements to access the data were signed by the authors, and data were subsequently deidentified and transferred to the authors in a password-protected file. For the present study (project number: CC-040), caregiver-reported questionnaire data were extracted for mothers and fathers/partners of children with CL/P who contributed data at both time points.

### Outcome measures

The *Pediatric Quality of Life Inventory—Family Impact Module* (PedsQL-FIM; Varni et al., 2004) is a 36-item measure of the impact of the child’s health on the family’s QoL and is divided into eight subscales: the caregiver’s physical, emotional, social, and cognitive functioning, their communication with others, and their worry for their child, and the impact on the family’s daily activities and relationships. Items are rated on a 5-point scale (0 = *never*; 4 = *almost always*) where higher scores indicate better functioning. A total score, health-related QoL summary score, and family functioning summary score were calculated, in addition to the eight subscale scores. The *Perceived Stress Scale* (PSS-10; Cohen et al., 1983) is a 10-item measure of perceived stress during the past month. Items are rated on a 5-point scale (0 = *never*; 4 = *very often*), where a higher score indicates a higher level of perceived stress. The *Hospital Anxiety and Depression Scale* (HADS; Zigmond & Snaith, 1983) is a 14-item measure of common anxiety and depression symptoms during the past month. The measure consists of seven anxiety questions (HADS-A) and seven depression questions (HADS-D), rated on a 4-point scale (e.g., 0 = *not at all*; 3 = *most of the time*). Higher scores indicate greater anxiety/depression.

### Predictor variables

The *Pediatric Quality of Life Inventory—Healthcare Satisfaction Generic Module* (PedsQL-HSGM; Varni et al., 2004) is a 24-item measure assessing six dimensions of healthcare satisfaction (Information, Inclusion of Family, Communication, Technical Skills, Emotional Needs, and Overall Satisfaction). Items are rated on a 5-point scale (0 = *never*; 4 = *almost always*) where a higher score indicates greater satisfaction. The *Revised Life Orientation Test* (LOT-R; Scheier et al., 1994) is a 10-item measure of optimism and pessimism. Items are rated on a 5-point scale (0 = *strongly disagree*; 4 = *strongly agree*) where a higher score indicates a more positive life orientation. The *Social Readjustment Rating Scale* (SRRS; Holmes & Rahe, 1967) is a 43-item measure of stressful life events occurring in the last year. A total score of 300 or more indicates a high risk of developing a stress-related illness, a score of 150–299 indicates a moderate risk, and a score of <150 indicates a mild risk. The *Relationship Satisfaction Scale* (RS10; Røysamb et al., 2014) is a 10-item measure of an individual’s subjective satisfaction with their relationship with their current partner. Participants who are in a relationship at the time of questionnaire completion respond using a 6-point scale (0 = *strongly disagree*; 5 = *strongly agree*) where a lower score indicates a higher level of satisfaction. The *Clinical Excellence Network Questionnaire* (CEN-Q; baseline version; Stock et al., 2016) is a 7-item condition-specific measure that reflects the degree to which caregivers appraise their child’s cleft negatively. The CEN-Q is a non-validated instrument, designed in accordance with existing literature, clinical input, and public involvement specifically for The Cleft Collective. Items are rated on a 5-point scale (0 = *never*; 4 = *almost always*) and a higher score indicates more negative appraisals.

### Analysis

Review, verification, and validation of the dataset were undertaken prior to descriptive and inferential analysis. Mean scores were calculated for each outcome measure and relevant subscales. Reliability was assessed using Cronbach’s alpha. There were no unduly large or strongly influential observations in the sample. Normative scores for the PedsQL-FIM, PSS-10, and HADS were derived from Medrano et al. (2013), Cohen (1988), and Crawford et al. (2001), respectively. Clinical cut-off scores for the current sample were estimated for PedsQL-FIM using published data from Medrano et al. (2013). It was estimated that approximately 5% of those in a non-clinical population would score <60 on the Total PedsQL-FIM, and approximately 25% would score between 61 and 79. A score of 80–100 was therefore tentatively considered “normal,” 61–79 was “borderline,” and a score <60 was “clinically concerning.” A PSS-10 score of 14–26 was “moderate perceived stress,” and 27–40 was “high perceived stress” (Cohen et al., 1983). A HADS score of 0–7 was “normal,” 8–10 was “borderline,” and 11 was “clinically concerning” (Zigmond & Snaith, 1983). A crosstabulation assessed differences in scores for each outcome measure across the timepoints. The McNemar–Bowker test was used to assess the statistical significance of changes in ordered categorical measures. Paired samples *t*-tests were used to compare T1 and T2 outcome scores. The independent samples *t*-test was used to compare sample scores against normative data. Effect size was calculated using Cohen’s *d*. Mothers and fathers/partners were analyzed separately based on known gender-specific differences in parenting experiences (e.g. Neumann et al., 2024), and the findings of the prior CL/P baseline study (Stock et al., 2020).

A series of exploratory analyses were performed to determine eligible variables for inclusion in the regression models. Pearson correlation coefficient (*r*) was calculated as an index of strength between potential baseline predictors and outcome variables at 5 years. Variables were included in the regression models provided that there was some evidence of an effect (*p* ≤ .05) and their inclusion did not violate the core assumptions of the regression test. For mothers, variables that were significantly correlated with at least one 5-year outcome included corresponding baseline measures, the five baseline standardized predictor measures (LOT-R, CEN-Q, PedsQL-HSGM, SRRS, and RS10), and single-item psychological and biodemographic data, as follows: illness during pregnancy, number of parent-reported mental health conditions at baseline; and age at conception. For fathers/partners, variables that were significantly correlated with at least one 5-year outcome included corresponding baseline measures, two baseline standardized measures (LOT-R and CEN-Q), and single-item data including the number of parent-reported mental health conditions. Irrespective of their statistical significance, the child’s cleft type and gender were retained in the multivariable models as control variables. For each regression model, the Benjamini–Hochberg procedure was used to control the false discovery rate at 0.1 (i.e., no more than 10% of positive findings to be false).

## Results

### Participant characteristics

The sample comprised 525 caregivers (342 biological mothers, 183 fathers/partners) of children born with CL/P who contributed both T1 and T2 data to The Cleft Collective dataset between December 2013 and December 2022 (Table 1).

**Table 1.** Sample characteristics (acquired from self-report “baseline” questionnaires).

Caregiver characteristics	Mothers (*n* = 342)		Fathers/partners (*n* = 183)		UK Census Data
**Mean (SD) age at conception**	31.43 (5.978)		34.40 (7.136)		
Median age at conception	32		34		
	IQR = 28–34		IQR = 30–38		
**Annual gross income**					£28 677
0–19,999	173	50.6%	40	21.9%	
20,000–39,999	114	33.4%	91	49.7%	
40,000–59,999	11	3.2%	27	14.7%	
60,000+^a^	8	2,3%	21	11.5%	
Missing	36	10.5%	4	2.2%	
**Education**					
No qualifications^a^	–	–	–	–	22.7%
School-level qualifications	117	34.2%	64	35.0%	40.9%
Undergraduate degree or above	177	51.7%	82	44.8%	27.2%
Other^a^	38	11.2%	37	20.2%	9.3%
Missing	10	2.9%	–	–	
**Country of birth**					
United Kingdom	275	80.4%	150	82.0%	87%
Other	54	15.8%	30	16.4%	13%
Missing	13	3.8%	3	1.6%	
**Ethnicity**					
White	304	88.9%	170	92.9%	86.0%
Mixed^a^	–	–	–	–	2.2%
Asian or Asian British^a^	–	–	–	–	7.5%
Black or Black British^a^	–	–	–	–	3.3%
Chinese or Chinese British^a^	–	–	–	–	
Other Mixed Background^a^	25	7.3%	7	3.8%	1.0%
Missing	13	3.8%	6	3.3%	
**Marital status**					
Domestic partner, married or in a civil union	311	90.9%	176	96.2%	46.7%
Single^a^	25	7.3%	5	2.7	34.7%
Separated or divorced^a^	6	1.8%	2	1.1%	11.6%
**Child characteristics (reported by mothers at baseline)**					
**Cleft type**					
Cleft palate only	135	39.5%			43%
Cleft lip only	83	24.3%			22%
Cleft lip + palate	124	36.2%			35%
**Mean (SD) child age (months) at baseline questionnaire completion**	6.52 (3.98)				
	IQR = 3.4–9.5				

*Note*. IQR = interquartile range.

a Due to the small number of participants in these categories, data were aggregated where possible to mitigate the risk of disclosure.

There was no notable presence of missing demographic data (income, education, country of birth, ethnicity, and marital status; Supplementary Table S1). Compared to UK Census data (Office for National Statistics, 2012), the sample was found to be predominantly White, UK-born and educated. Participants also reported above-average median household income for two-parent families (Office for National Statistics, 2018). The distribution of the child’s cleft type was found to be relatively comparable with national audit data (CRANE, 2019).

### Utility of outcome measures

The PedsQL-FIM demonstrated excellent internal reliability (α = .98 for mothers and α = .97 for fathers/partners). Internal consistency was also robust across the different domains (α  =  .80–.97 for mothers and fathers/partners). The PSS-10 demonstrated good internal reliability (α = .87 for mothers and fathers/partners), consistent with previous studies (Cohen et al., 1983). The HADS-A (α = .86 for mothers and α = .89 for fathers/partners) and HADS-D (α = .84 for mothers and α = .86 for fathers/partners) also demonstrated good internal reliability. All outcomes were significantly correlated with one another (Supplementary Table S2).

### Comparisons to normative data at 5 years

PedsQL-FIM scores were significantly higher than norms for mothers (*t*(551) = 12.160, *p* < .001) and fathers/partners (*t*(273) = 12.698, *p* < .001; Table 2), suggesting higher QoL. The mean difference between mothers’ scores and norms was 13.40 [95% confidence interval (CI) 11.24–15.57], with a large effect size (*d *= 1.04). The mean difference between fathers’/partners’ scores and norms was 15.60 (95% CI 13.18–18.02), with a large effect size (*d *= 1.54). Fathers/partners reported significantly less anxiety than the general population on the HADS-A (*t*(214) = 4.161, *p* < .001). The mean difference was 1.32 (95% CI 0.70–1.95), with a medium effect size (*d *= .57). No statistically significant differences were found between mothers’ anxiety (HADS-A) and normative data, or between mothers’ or fathers’/partners’ depression (HADS-D) and stress (PSS-10) scores compared to normative data.

**Table 2.** Comparison of 5-year outcomes to normative data.

Outcome measure	Normative mean (SD)	Mothers’ mean (SD)	Mothers' independent samples *t*-test	Cohen’s *d*	Fathers’/partners’ mean (SD)	Fathers'/partners independent samples *t*-test	Cohen’s *d*
**PedsQL-FIM**	70.8 (14.5)	84.2 (17.6)	12.160***	1.04	86.4 (14.9)	12. 698***	1.54
HRQoL summary	69.4 (15.5)	81.9 (19.8)	10.356***	0.91	84.2 (16.7)	10.968***	1.37
Family functioning	65.5 (18.5)	88.8 (19.4)	18.939***	1.57	90.3 (16.3)	18.217***	2.15
Physical functioning	64.9 (17.4)	78.7 (23.0)	10.009***	0.91	81.7 (18.4)	11.363***	1.43
Cognitive functioning	73.5 (18.6)	84.4 (21.2)	8.266***	0.71	85.1 (18.2)	7.773***	0.96
Emotional functioning	67.6 (17.9)	81.3 (20.9)	10.667***	0.92	84.5 (19.5)	10.830***	1.38
Social functioning	74.4 (19.1)	84.5 (21.4)	7.617***	0.65	86.4 (18.9)	7.813***	0.96
Worry for child	78.1 (20.1)	83.5 (20.6)	4.104***	0.34	86.6 (18.6)	5.518***	0.67
Communication	81.9 (17.7)	86.3 (20.8)	3.438***	0.30	90.5 (16.9)	6.193***	0.76
Family relationships	67.0 (19.4)	89.8 (18.9)	18.657***	1.50	90.6 (17.0)	16.625***	1.96
Daily activities	63.2 (22.5)	87.7 (22.5)	17.035***	1.40	89.8 (19.1)	16.605***	1.94
**HADS-A**	6.14 (3.76)	6.20 (4.12)	0.250	0.02	4.82 (4.12)	4.161***	0.57
**HADS-D**	3.68 (3.07)	3.80 (3.56)	0.583	0.05	3.35 (3.41)	1.258	0.17
**PSS-10**	Females 13.7 (6.6)	14.2 (7.3)	1.151	0.11	–	–	
	Males 12.1 (5.9)	–	–		13.0 (7.1)	1.605	0.21

*Note*. CEN-Q = Clinical Excellence Network Questionnaire; HADS-A = Hospital Anxiety and Depression Scale—Anxiety; HADS-D = Hospital Anxiety and Depression Scale—Depression; HRQoL = health-related quality of life; PedsQL-FIM = Pediatric Quality of Life Inventory—Family Impact module; PSS-10 = Perceived Stress Scale; SD = standard deviation.

*** *p* < .001 (2 tailed).

** *p* < .01 (2 tailed).

* *p* < .05 (2 tailed).

### Changes in outcomes over time

There was a statistically significant increase in mothers’ PedsQL-FIM mean scores from T1 to T2 (*t*(333) = 8.979, *p* < .001. The mean increase was 9.33 (95% CI 7.28–11.37) with a medium standardized effect size (*d *= .53). For anxiety, depression, and stress, no statistically significant changes in mothers’ and fathers’/partners’ mean scores from T1 to T2 were identified. Fathers also showed a statistically significant increase in PedsQL-FIM scores from T1 to T2 (*t*(180) = 7.647, *p* <. 001; Table 3), indicating improvements in QoL. The mean increase was 9.03 (95% CI 6.70–11.36), with a medium standardized effect size (*d *= .57).

**Table 3.** Changes in mean outcome scores between T1 and T2.

Outcome measure	Mothers’ T1 (SD)	Mothers’ T2 (SD)	Mothers' dependent samples *t*-Test	Cohen’s *d*	Fathers’/partners’ T1 (SD)	Fathers’/partners’ T2 (SD)	Fathers'/partners’ dependent samples *t*-test	Cohen’s *d*
**PedsQL-FIM**	74.86 (17.14)	84.19 (17.64)	8.98***	.54	77.31 (15.52)	86.34 (14.93)	7.65***	.58
HRQoL summary	72.61 (18.20)	81.82 (19.75)	7.77***	.51	75.44 (16.36)	84.41 (16.53)	6.40***	.55
Family functioning	81.06 (20.27)	88.70 (19.49)	5.82***	.38	80.46 (19.55)	90.52 (15.93)	6.16***	.52
Physical functioning	66.67 (20.47)	78.30 (23.03)	7.41***	.57	70.28 (18.20)	81.76 (18.32)	6.70***	.63
Cognitive functioning	74.21 (21.78)	84.39 (21.26)	7.49***	.47	75.17 (20.44)	85.01 (18.18)	6.26***	.48
Emotional functioning	72.69 (21.54)	81.27 (20.66)	6.78***	.40	78.03 (19.65)	84.64 (19.45)	4.0***	.34
Social functioning	78.05 (21.25)	84.77 (21.20)	5.02***	.32	80.16 (19.06)	86.75 (18.82)	3.89***	.35
Worry for child	71.34 (19.92)	83.40 (20.63)	9.97***	.61	75.65 (20.39)	86.51 (1.39)	6.95***	.53
Communication	78.37 (22.26)	86.31 (20.82)	6.31***	.36	82.24 (18.69)	90.42 (16.93)	5.16***	.44
Family relationships	85.62 (19.68)	89.66 (19.03)	3.15**	.21	84.18 (19.13)	90.75 (16.81)	4.01***	.34
Daily activities	73.39 (26.43)	87.61 (22.54)	8.46***	.54	74.12 (25.24)	90.08 (18.65)	7.48***	.63
**HADS-A**	6.36 (4.05)	6.20 (4.12)	.80	.04	5.23 (3.76)	4.81 (4.12)	1.44	.11
**HADS-D**	4.15 (3.32)	3.80 (3.56)	1.82	.11	3.56 (3.56)	3.35 (3.41)	.79	.06
**PSS-10**	14.44 (7.12)	14.26 (7.29)	.48	.03	12.82 (7.33)	12.97 (7.13)	.27	.02

*Note*: CEN-Q = Clinical Excellence Network Questionnaire; HADS-A = Hospital Anxiety and Depression Scale—Anxiety; HADS-D, Hospital Anxiety and Depression Scale—Depression; HRQoL = health-related quality of life; PedsQL-FIM = Pediatric Quality of Life Inventory—Family Impact module; PSS-10 = Perceived Stress Scale; SD = standard deviation.

*** *p* < .001 (2 tailed).

** *p* < .01 (2 tailed).

* *p* < .05 (2 tailed).

### Caregiver appraisals

More negative appraisals of CL/P (CEN-Q) were associated with greater anxiety, depression, perceived stress, and QoL impact scores for mothers and fathers/partners at both time points (Supplementary Table S4). Caregiver appraisals became significantly more positive from T1 to T2 for both mothers and fathers/partners (Supplementary Table S3). For mothers, the change was significant (*t*(305) = 8.916, *p* < .001), with a mean difference of 2.24 (95% CI 1.75–2.74) and a medium standardized effect size (*d *= .51). These positive changes in CL/P appraisals were also significantly correlated with the changes observed in mothers’ outcomes scores over time, including HADS-A (*r* = .27), HADS-D (*r* = .28), PSS-10 (*r* = .28), and PedsQL-FIM (*r *= −.33). For fathers/partners, the change was also significant (*t*(168) = 4.416, *p* < .001), with a mean difference of 1.28 (95% CI 0.71–1.86) and a small standardized effect size (*d *= .36). Fathers/partners’ changes in CL/P appraisals were also significantly correlated with changes observed over time in their HADS-A (*r* = .22), HADS-D (*r* = .25), and PedsQL-FIM (*r *= −.35). However, the correlations between changes in fathers/partners’ appraisals were not significantly associated with changes in their PSS-10 scores over time. Additional analysis revealed these changes in CEN-Q scores to be significantly correlated with improvements in anxiety, depression and QoL in both mothers and fathers/partners, and perceived stress in mothers (Supplementary Table S4).

### Classification of scores

From T1 to T2, the percentage of caregivers in the “normal,” “borderline,” and “concern” categories remained stable in relation to stress, anxiety, and depression, with no statistically significant changes occurring over time (Table 4). On the PedsQL-FIM, percentages generally improved, with fewer caregivers falling into the borderline and clinical categories at T2 for both mothers (*χ^2^*(3) = 55.571, *p* < .001) and fathers/partners (*χ^2^*(3) = 28.129, *p* < .001).

**Table 4.** Classification of Scores at T1 and T2.

		T1				T2			
Mothers	Measure	*N*	Normal	Borderline	Concern	*N*	Normal	Borderline	Concern
	PedsQL-FIM	341	42.3%	37.2%	20.5%	335	66.9%	20.6%	12.5%
	PSS-10	337	45.4%	49.5%	5.1%	338	47.6%	46.8%	5.6%
	HADS-A	342	62.3%	21.6%	16.1%	341	68.3%	14.7%	17.0%
	HADS-D	342	81.6%	14.3%	4.1%	342	84.2%	12.0%	3.8%
Fathers/partners	Peds_QL-FIM	182	47.2%	39.6%	13.2%	182	68.7%	24.7%	6.6%
	PSS-10	183	57.1%	38.0%	4.9%	182	51.1%	46.7%	2.2%
	HADS-A	183	73.8%	18.0%	8.2%	183	76.5%	12.6%	10.9%
	HADS-D	183	86.9%	8.2%	4.9%	183	89.1%	6.0%	4.9%

### Predictors of maternal well-being at 5 years

Exploratory analyses determined eligible variables for inclusion in mothers’ regression models (Supplementary Table S5).

#### Family quality of life

The regression model accounted for 28.7% of the variance in mothers’ scores and comprised two statistically significant variables (adjusted *R*^2^ = .247, *F*(12,213) = 7.161; *p* < .001; Table 5). Lower healthcare satisfaction and more negative appraisals of CL/P at T1 were associated with poorer family QoL at T2. However, this borderline significant effect on healthcare satisfaction would not be considered significant under the Benjamini–Hochberg procedure with False Discover Rate = .1.

**Table 5.** Regression models for mothers and fathers/partners at 5 years.

Mothers			
T2 outcome measures and T1 predictor variables	β	*t*	*p*-value
**PedsQL-FIM (*n* = 226)**			
LOT-R	.058	0.876	.382
CEN-Q	−.313	−4.342	<.001
PedsQL-HSGM	.148	2.403	.017
Illness during pregnancy	−.084	−1.374	.171
Number of mental health conditions	−.111	−1.707	.089
Age at conception	.082	0.940	.348
Relationship satisfaction	.081	1.255	.211
Social Readjustment Scale	.012	0.181	.856
Gender: girl	−.024	−0.395	.693
Cleft lip only	.070	1.028	.305
Cleft palate only	−.098	−1.431	.154
PedsQL-FIM at baseline	.069	0.940	.348
**PSS-10 (*n* = 225)**			
LOT-R	−.173	−2.492	.013
CEN-Q	.053	0.730	.466
PedsQL-HSGM	−.162	−2.598	.010
Illness during pregnancy	−.007	−0.114	.909
Number of mental health conditions	.048	0.709	.479
Age at conception	−.054	−0.855	.394
Relationship satisfaction	−.031	−0.472	.637
Social Readjustment Scale	.028	0.422	.674
Gender: girl	.028	0.451	.653
Cleft lip only	−.046	−0.672	.502
Cleft palate only	−.058	−0.824	.411
PSS at baseline	.276	3.545	<.001
**HADS-A (*n* = 230)**			
LOT-R	−.140	−2.324	.021
CEN-Q	.076	1.144	.254
PedsQL-HSGM	−.143	−2.592	.010
Illness during pregnancy	−.030	−0.535	.593
Number of mental health conditions	.028	0.443	.658
Age at conception	.004	0.075	.940
Relationship satisfaction	−.054	−0.932	.352
Social Readjustment Scale	.037	0.633	.528
Child gender	.053	0.974	.331
Cleft lip only	−.143	−2.329	.021
Cleft palate only	−.155	−2.476	.014
HADS-D at baseline	.419	5.961	<.001
**HADS-D (*n* = 230)**			
LOT-R	−.121	−1.764	.079
CEN-Q	.084	1.210	.228
PedsQL-HSGM	−.198	−3.217	.001
Illness during pregnancy	−.033	−0.531	.596
Number of mental health conditions	.025	0.365	.715
Age at conception	−.039	−0.628	.531
Relationship satisfaction	−.069	−1.081	.281
Social Readjustment Scale	−.017	−0.267	.790
Child gender	.013	0.213	.832
Cleft lip only	−.013	−0.194	.846
Cleft palate only	−.046	−0.656	.512
HADS-D at baseline	.291	4.104	<.001

Fathers			
T2 outcome measures and T1 predictor variables	β	*t*	*p*-value
**PedsQL-FIM (*n* = 178)**			
LOT-R	−.062	−0.870	.385
CEN-Q	−.161	−2.109	.036
Number of mental health conditions	−.216	−3.237	.001
Cleft lip only	.047	0.617	.538
Cleft palate only	−.111	−1.405	.162
Gender: girl	.029	0.427	.670
PedsQL-FIM at baseline	.382	4.852	<.001
**PSS-10 (*n* = 178)**			
LOT-R	−.100	−1.427	.155
CEN-Q	.053	0.757	.450
Number of mental health conditions	.213	3.250	.001
Cleft lip only	.089	1.190	.236
Cleft palate only	.109	1.415	.159
Gender: girl	−.020	−0.302	.763
PSS-10 at baseline	.430	5.850	<.001
**HADS-A (*n* = 180)**			
LOT-R	−.201	−2.906	.004
CEN-Q	.033	0.481	.631
Number of mental health conditions	.201	3.065	.003
Cleft lip only	.066	0.891	.374
Cleft palate only	.096	1.255	.211
Gender: girl	−.011	−0.173	.863
HADS-A at baseline	.376	5.138	<.001
**HADS-D (*n* = 180)**			
LOT-R	−.076	−1.108	.269
CEN-Q	.033	0.484	.629
Number of mental health conditions	.242	3.738	<.001
Cleft lip only	.149	2.009	.046
Cleft palate only	.187	2.451	.015
Gender: girl	−.014	−0.212	.832
HADS-D at baseline	.442	6.190	<.001

*Note*. CEN-Q: Clinical Excellence Network Questionnaire; HADS-A: Hospital Anxiety and Depression Scale—Anxiety; HADS-D: Hospital Anxiety and Depression Scale—Depression; LOT-R: Revised Life Orientation Test; PedsQL-HSGM: Pediatric Quality of Life Inventory—Healthcare Satisfaction Generic Module; PedsQL-FIM: Pediatric Quality of Life Inventory—Family Impact module; PSS-10: Perceived Stress Scale; SD: standard deviation.

#### Perceived stress

The regression model accounted for 25.8% of the variance in mothers’ scores and comprised three statistically significant variables (adjusted *R*^2^ = .216, *F*(12,212) = 6.141; *p* < .001). A less positive life orientation, lower healthcare satisfaction, and higher perceived stress at T1 were associated with higher perceived stress at T2.

#### Anxiety

The regression model accounted for 39.7% of the variance in mothers’ scores and comprised three statistically significant variables (adjusted *R*^2^ = .364, *F*(12,217) = 11.928; *p* < .001). A less positive life orientation, lower healthcare satisfaction, and higher anxiety at T1 were associated with higher anxiety at T2. After controlling for T1, mothers of children with combined cleft lip and palate reported greater anxiety at T2 than mothers whose children had a cleft lip/cleft palate only.

#### Depression

The regression model accounted for 24.7% of the variance in mothers’ scores and comprised two statistically significant variables (adjusted *R*^2^ = .206, *F*(12,217) = 5.940; *p* <.001). Lower healthcare satisfaction and higher depression scores at T1 were associated with higher levels of depression at T2.

### Predictors of paternal well-being at 5 years

Exploratory analyses determined eligible variables for inclusion in fathers’ regression models (Supplementary Table S6).

#### Family quality of life

The regression model accounted for 28.7% of the variance in fathers’/partners’ scores and comprised three statistically significant variables (adjusted *R*^2^ = .258, *F*(7,170) = 9.782; *p* < .001; Table 5). More negative appraisals of CL/P, a higher number of parent-reported prior mental health conditions, and a greater impact on family QoL at T1 were associated with poorer family QoL at T2.

#### Perceived stress

The regression model accounted for 32.3% of the variance in fathers’/partners’ scores and comprised two statistically significant variables (adjusted *R*^2^ = .295, *F*(7,170) = 11.596; *p* < .001). A higher number of parent-reported prior mental health conditions and higher perceived stress at T1 were associated with higher perceived stress at T2.

#### Anxiety

The regression model accounted for 32.3% of the variance in fathers’/partners’ scores and comprised three statistically significant variables (adjusted *R*^2^ = .296, *F*(7,172) = 11.731; *p* < .001). Higher anxiety, a less positive life orientation, and a higher number of parent-reported prior mental health conditions at T1 were associated with higher anxiety at T2.

#### Depression

The regression model accounted for 32.7% of the variance in fathers’/partners’ scores and comprised four statistically significant variables (adjusted *R*^2^ = .300, *F*(7,172) = 11.958; *p* < .001). A higher number of parent-reported prior mental health conditions and higher depression scores at T1 were associated with higher depression scores at T2. After controlling for T1, fathers/partners of children with combined cleft lip and palate reported greater depression at T2 than fathers/partners whose children had a cleft lip/cleft palate only.

## Discussion

### Overall synthesis of findings

This study investigated longitudinal psychological well-being in caregivers of young children with CL/P from “baseline” (diagnosis) to 5 years. Overall, caregivers reported psychological well-being consistent with population norms at 5 years. Although the changes in caregiver-reported stress, anxiety, and depression from T1 to T2 were not significant, scores were in line with or better than general population norms at age 5. This is in contrast to our previous cross-sectional paper which found elevated distress in caregivers at “baseline” (Stock et al., 2020). Impact on family QoL significantly improved from T1 to T2 on all subscales, and fewer participants were categorized as ‘borderline’ or ‘clinically concerning’ at T2. These findings align with prior cross-sectional and qualitative CL/P studies that identify the first year of the child’s life to be particularly distressing for caregivers (Stock et al., 2024).

The few general population studies conducted in this area suggest that parents of younger children may have enhanced well-being compared to parents of older children (e.g., Luthar & Ciciolla, 2016; Nomaguchi, 2012; Pollmann-Schult, 2014; Roeters & Gracia, 2016). While attributional causality cannot be assumed, the findings of the current study may therefore point to a positive effect of multidisciplinary cleft care on caregiver well-being. The centralization of CL/P services in the early 2000s has resulted in highly specialized teams with the ability to assess and monitor the holistic needs of the family throughout the treatment journey, allowing for a proactive approach to intervention. In addition, the Cleft Lip and Palate Association offers complementary UK-wide peer support services and events for families from diagnosis onwards (Stock et al., 2020). Further research to assess the contribution of these and other aspects of care in improving caregiver outcomes could be valuable for informing CL/P and other healthcare services.

### The role of caregiver appraisals

Caregiver appraisals can greatly influence how well a family adjusts to challenging situations (McCubbin & McCubbin, 1996). In line with this, negative appraisals of CL/P were associated with negative outcomes at baseline and 5 years and predicted poorer QoL at 5 years. This builds upon previous research highlighting associations between parental appraisals of their child’s cleft and parental well-being (Shuttlewood et al., 2014). Furthermore, reductions in CEN-Q scores (which reflects the degree to which caregivers negatively appraise their child’s cleft) were associated with improvements in caregiver outcomes from T1 to T2. McCubbin and McCubbin (1996) also demonstrated the role of adequate support and resources in positively influencing caregiver appraisals. Prior research has shown that once a family is under the care of a specialist CL/P team and has access to comprehensive information and support, caregiver well-being and healthcare satisfaction tend to improve (Stock et al., 2024). Increases in caregiver well-being have also been documented following the completion of the primary surgeries (Macho et al., 2017). It could therefore be that at 5 years, caregivers’ initial concerns have largely been resolved, they have learnt that previous appraisals were perhaps inaccurate or overly negative, they feel well supported, and the current treatment burden is relatively low. Targeting caregiver appraisals from an early stage may therefore help to facilitate caregiver and family QoL. MDTs could initiate exploratory conversations to identify and normalize any negative appraisals and provide education and reassurance (e.g., Sood et al., 2023; Stock et al., 2020). Education and training for MDTs to enable such conversations may be warranted. In addition, printed or online psychoeducational materials for families that normalize concerns, target appraisals, and signpost families to various sources of support may be beneficial (e.g., Stock et al., 2022), alongside putting caregivers in touch with peer support networks (Lancaster et al., 2023). For caregivers who hold negative appraisals that are particularly rigid or resistant to change, specialist psychologists can offer targeted psychological interventions (e.g., cognitive behavior therapy or acceptance and commitment therapy; Beck, 1967, 1976; Hayes et al., 1999). Given the predictive value of baseline negative appraisals on family QoL 5 years later, the early identification of, and interventions for, such appraisals seem particularly important. Future research could seek to develop or adapt psychological interventions aimed at improving specific parental appraisals. Additional research could also examine whether parental appraisals of CL/P can impact the child.

### Identifying caregivers at high risk of distress

Despite positive overall findings, a minority of caregivers reported scores considered to be clinically concerning at five years on at least one outcome measure. A consistent predictor of poorer outcomes at T2 was outcomes at T1. This further emphasizes the need to screen caregivers following a diagnosis and discuss psychological well-being at regular intervals to enable early intervention (Salley et al., 2024). The measures used in this study appear to have clinical utility. Specifically, the HADS and PSS-10 were able to identify psychological distress in caregivers, while the PedsQL-FIM picked up cleft-related concerns. These measures have been recommended for use in global CL/P initiatives (Stock et al., 2016). Similar and additional measures were also proposed by the Americleft Psychosocial Outcomes project (Crerand et al., 2017) and a craniofacial screening tool was introduced in 2018 (Psychosocial Assessment Tool—Craniofacial Version; Crerand et al., 2018).

Research-informed psychological interventions to support caregivers of children with CL/P are scarce and additional research to build this evidence base is critical. Specific interventions, such as the Promoting Resilience In Stress Management (PRISM) program may be effective in mitigating distress and enhancing resiliency among caregivers of children with CL/P and other chronic conditions (Fladeboe et al., 2024; Yi-Frazier et al., 2017). However, prolonged distress (anxiety, depression, stress) may or may not be associated with CL/P. It was not possible within the current study to determine whether parental distress at T2 was related to the child’s cleft or to other factors. MDTs could seek to establish the cause of parental distress in order to consider which support or treatment pathway(s), including signposting to additional services, may be most appropriate.

### Additional predictors of caregiver well-being

Healthcare satisfaction was a significant predictor of maternal well-being. Many studies have demonstrated the influence of healthcare quality on caregiver outcomes, with a particular emphasis on interpersonal care (Batbaatar et al., 2017; Stock et al., 2020). While overall ratings of healthcare satisfaction tend to be high, a better understanding of the different components that contribute to satisfaction in cleft care settings would be beneficial (Costa et al., 2020). Cleft teams could also engage in quality improvement processes, including monitoring and evaluating caregiver feedback, to positively affect caregivers’ perceptions and experiences of their child’s cleft care. Also in line with prior research (Cousino & Hazen, 2013; Stock et al., 2020), the existence of parent-reported prior mental health conditions predicted paternal well-being, indicating that early assessment to flag this risk factor is warranted. Finally, caregivers of children with combined cleft lip and palate (as opposed to other cleft types, such as cleft lip only) reported poorer outcomes, which may be explained in part by greater perceived child vulnerability and a higher anticipated treatment burden (Thomas et al., 2024; Thompson et al., 2021).

### Methodological considerations

This study utilized a national CL/P birth cohort to present novel longitudinal data on caregiver well-being during the first five years of their child’s life. The findings provide further support for the integration of specialist psychologists and other key support staff (e.g. clinical nurse specialists; Searle et al., 2018) in MDTs and the inclusion of early caregiver assessment and support. Nonetheless, the study sample may be limited in its representativeness. First, and despite highly inclusive eligibility criteria, caregivers from diverse ethnic and cultural backgrounds, those having immigrated to the UK, and those with lower socioeconomic status are underrepresented in the current sample compared to national averages. Broader health literature has demonstrated clear differences in the way these groups interact with health services and engage with research (Public Health England, 2017). Several CL/P-focused studies have also been indicative of poorer outcomes among these subgroups (Stock & Feragen, 2016). Continued efforts are needed to ensure that studies are relevant and accessible to eligible participants (Zucchelli et al., 2018). The percentage of fathers/partners providing data was also comparatively low, which may relate to the primary caregiver role. Additionally, a higher percentage of fathers/partners were married or in domestic partnerships, suggesting that non-absent fathers/partners are more inclined or have more opportunities to participate in data collection. Future studies could delve deeper into the role that family type plays in the profile of caregivers (Toledano-Toledano and Luna, 2020). Data on syndromic status were unavailable at the time of data analysis, limiting the ability to assess the role of this characteristic. Some participants also declined to complete some measures, resulting in a reduction in sample size during the regression analyses. Caution is also advised regarding the use of multiple hypothesis testing, which was controlled for in the current analyses using the Benjamini–Hochberg procedure.

## Conclusions

This study demonstrated a significant improvement in family QoL over time for both mothers and fathers/partners. At 5 years, scores on all measures of psychological distress were in line with/more favorable than norms. Yet, a minority of caregivers continued to report clinically significant levels of distress at five years. This study lends further support to the need for routine assessment and intervention for caregivers of children with chronic health conditions, including CL/P. Identifying and modifying early negative cleft-related appraisals may also help to facilitate caregiver QoL. Further work is needed to understand how best to screen caregivers in a busy clinical environment to ensure the most vulnerable families are identified and offered support.

## Supplementary Material

jsaf029_Supplementary_Data

## Data Availability

The data underlying this article were provided by The Cleft Collective by permission. Data will be shared on request to the corresponding author with permission of The Cleft Collective research team.

## Artificial intelligence

AI-assisted technologies have not been used in the writing process.
