# Supplementary material for: Longitudinal psychological well-being in caregivers of young children with cleft lip and/or palate
Source: J Pediatr Psychol. 2025 May 7;50(9):831–42. doi: 10.1093/jpepsy/jsaf029 (PMC12451232; doi:10.1093/jpepsy/jsaf029)
Supplement: jsaf029_Supplementary_Data [file jsaf029_supplementary_data.docx]

**SUPPLEMENTARY MATERIALS**

| **Suppl. Table 1:** Missing Data Analysis | | | | |
| --- | --- | --- | --- | --- |
| Measure | Group 0 (SD) | Group 1 (SD) | Independent samples t test | Cohen’s d |
| Mothers | | | | |
| HADS-A | 6.22 (4.17) | 6.11 (3.94) | 0.20 | 0.03 |
| HADS-D | 3.87 (3.64) | 3.51 (3.17) | 0.71 | 0.10 |
| PSS-10 | 14.31 (7.46) | 13.48 (6.76) | 0.79 | 0.11 |
| PedsQL-FIM | 84.07 (17.93) | 84.98 (16.38) | -0.37 | -0.05 |
| Fathers/Partners | | | | |
| HADS-A | 4.78 (4.05) | 5.05 (4.75) | -0.28 | -0.06 |
| HADS-D | 3.38 (3.49) | 3.11 (2.80) | 0.34 | 0.08 |
| PSS-10 | 12.99 (7.08) | 12.91 (7.61) | 0.05 | 0.01 |
| PedsQL-FIM | 86.88 (14.46) | 82.63 (18.25) | 1.20 | 0.29 |

Abbreviations: Group 0, participants without missing demographic information; Group 1, participants with ≥1 missing demographic information.

* 0.01 < p < .05

** 0.001 < p < 0.01

*** p < .001

| **Suppl. Table 2:** Correlations Between Outcomes at 5 Years | | | | |
| --- | --- | --- | --- | --- |
|  | HADS-A | HADS-D | PSS-10 | PedsQL-FIM |
| Mothers |  |  |  |  |
| HADS-A | 1 | .71*** | .73*** | -.55*** |
| HADS-D | .71*** | 1 | .66*** | -.56*** |
| PSS-10 | .73*** | .66*** | 1 | -.53*** |
| PedsQL-FIM | -.55*** | -.56*** | -.53*** | 1 |
| Fathers/Partners |  |  |  |  |
| HADS-A | 1 | .72*** | .73*** | -.54*** |
| HADS-D | .72*** | 1 | .66*** | -.51*** |
| PSS-10 | .73*** | .66*** | 1 | -.56*** |
| PedsQL-FIM | -.54*** | -.51*** | -.56*** | 1 |

* 0.01 < p < .05

** 0.001 < p < 0.01

*** p < .001

| **Suppl. Table 3:** Changes in CEN-Q Scores from T1 to T2 | | | | | | | | |
| --- | --- | --- | --- | --- | --- | --- | --- | --- |
| Measure | Mothers’ T1 (SD) | Mothers’ T2 (SD) | Mothers' Dependent samples t Test | Cohen d | Fathers’/Partners’ T1 (SD) | Fathers’/Partners’ T2 (SD) | Fathers'/Partners’ Dependent samples t Test | Cohen’s d |
| CEN-Q | 6.80 (4.75) | 4.56 (4.10) | 8.92*** | 0.51 | 4.65 (3.88) | 3.37 (3.16) | 4.42*** | 0.36 |

* 0.01 < p < .05

** 0.001 < p < 0.01

*** p < .001

**Suppl. Table 4:** Associations Between CEN-Q and Outcomes at T1 and T2

| CEN-Q | HADS-A | HADS-D | PSS-10 | PedsQL-FIM |
| --- | --- | --- | --- | --- |
| Mothers at T1 | .49*** | .43*** | .50*** | -.56*** |
| Mothers at T2 | .44*** | .39*** | .45*** | -.58*** |
| Fathers/Partners at T1 | .40*** | .36*** | .41*** | -.51*** |
| Fathers/Partners at T2 | .34*** | .34*** | .34*** | -.55*** |

* 0.01 < p < .05

** 0.001 < p < 0.01

*** p < .001

| **Suppl. Table 5:** Preliminary Analysis for Mothers’ Regression Models | | | | |
| --- | --- | --- | --- | --- |
|  | HADS-A | HADS-D | PSS-10 | PedsQL-FIM |
| LOT-R | -.36*** | -.30*** | -.35*** | .25** |
| PedsQL-HSGM | -.28*** | -.30*** | -.26*** | .28*** |
| Relationship Satisfaction | -.17* | -.14* | -.16* | .13* |
| Age at Conception | .00 | -.07 | -.12* | .08 |
| Absence from Work > 2 Weeks | .05 | .04 | .03 | -.11 |
| CEN-Q at T1 | .31*** | .24*** | .24*** | -.42*** |
| Satisfaction with Close Friends | -.05 | -.10 | -.02 | .07 |
| Social Readjustment Scale | .14* | .05 | .12 | -.05 |
| Illness During Pregnancy | .03 | .02 | .04 | -.14* |
| Number of Illnesses During Pregnancy | .01 | .03 | -.00 | -.10 |
| Mental Health Condition | .20** | .12 | .19** | -.12 |
| Number of Mental Health Conditions | .22** | .13 | .18** | -.10 |
| HADS-A at T1 | .56*** | .33*** | .36*** | -.40*** |
| HADS-D at T1 | .38*** | .41*** | .25*** | -.25*** |
| PSS-10 at T1 | .49*** | .39*** | .43*** | -.37*** |
| PedsQL-FIM at T1 | -.41*** | -.34*** | -.31*** | .38*** |

* 0.01 < p < .05

** 0.001 < p < 0.01

*** p < .001

| **Suppl. Table 6:** Preliminary Analysis for Fathers’/Partners’ Regression Models | | | | |
| --- | --- | --- | --- | --- |
|  | HADS-A | HADS-D | PSS-10 | PedsQL-FIM |
| LOT-R | -.34*** | -.25** | -.27*** | .11 |
| PedsQL-HSGM | -.01 | .06 | -.02 | .06 |
| Relationship Satisfaction | -.07 | .03 | .05 | -.03 |
| Age at Conception | .00 | .05 | -.07 | .12 |
| Absence from Work > 2 Weeks | .07 | .04 | .05 | .05 |
| CEN-Q at T1 | .23** | .21** | .25** | -.36*** |
| Satisfaction with Close Friends | -.02 | -.19 | -.09 | -.15 |
| Social Readjustment Scale | .04 | .06 | .04 | -.11 |
| Mental Health Condition | .28*** | .24** | .24** | -.20* |
| Number of Mental Health Conditions | .31*** | .33*** | .30*** | -.25*** |
| HADS-A at T1 | .56*** | .40*** | .44*** | -.38*** |
| HADS-D at T1 | .34*** | .49*** | .35*** | -.24** |
| PSS-10 at T1 | .47*** | .40*** | .51*** | -.41*** |
| PedsQL-FIM at T1 | -.43*** | -.36*** | -.39*** | .46*** |

* 0.01 < p < .05

** 0.001 < p < 0.01

*** p < .001
